# Comparison of the Minimum Inhibitory and Mutant Prevention Drug Concentrations for Pradofloxacin and 7 Other Antimicrobial Agents Tested Against Swine Isolates of *Actinobacillus pleuropneumoniae* and *Pasteurella multocida*

**DOI:** 10.3390/molecules29225448

**Published:** 2024-11-19

**Authors:** Joseph M. Blondeau, Shantelle D. Fitch

**Affiliations:** 1 Departments of Biochemistry, Microbiology and Immunology, Pathology and Laboratory Medicine and Ophthalmology, University of Saskatchewan, Saskatoon, SK S7N 0W8, Canada; 2 Department of Clinical Microbiology, Royal University Hospital and Saskatchewan Health Authority, Saskatoon, SK S7N 0W8, Canada; shantelle.fitch@saskhealthauthority.ca

**Keywords:** *Actinobacillus pleuropneumoniae*, *Pasteurella multocida*, MIC/MPC, pradofloxacin

## Abstract

Pradofloxacin is a dual targeting, bactericidal fluoroquinolone recently approved for treating bacteria causing swine respiratory disease. Currently, an abundance of in vitro data does not exist for pradofloxacin. We determined the minimum inhibitory concentration (MIC) and mutant prevention concentrations (MPC) of pradofloxacin compared to ceftiofur, enrofloxacin, florfenicol, marbofloxacin, tildipirosin, tilmicosin and tulathromycin against swine isolates of *Actinobacillus pleuropneumoniae* and *Pasteurella multocida*. Overall, pradofloxacin had the lowest MIC and MPC values as compared to the other agents tested. For example, pradofloxacin MIC values for 50%, 90% and 100% of *A. pleuropneumoniae* strains were ≤0.016 µg/mL, ≤0.016 µg/mL and ≤0.016 µg/mL and for *P. multocida* were ≤0.016 µg/mL, ≤0.016 µg/mL and 0.031 µg/mL, respectively. The MPC values for 50%, 90% and 100% of *A. pleuropneumoniae* strains were 0.031 µg/mL, 0.063 µg/mL and 0.125 µg/mL and for *P. multocida* were ≤0.016 µg/mL, 0.031 µg/mL and 0.0.063 µg/mL, respectively. By MPC testing, all strains were at or below the susceptibility breakpoint. Based on MPC testing, pradofloxacin appears to have a low likelihood for resistance selection. This study represents the most comprehensive in vitro comparison of the above noted drugs and the first report for pradofloxacin and tildipirosin.

## 1. Introduction

Swine respiratory disease complex is a multifactorial process giving rise to lung infection in pigs. Bacterial pathogens colonizing pigs are opportunistic and give rise to pulmonary infection following primary infection by viral agents such as porcine reproduction and respiratory syndrome virus (PRRSV), swine influenza virus (SIV), pseudorabies virus (PRV), porcine respiratory coronavirus (PRCV) and porcine circovirus type 2 (PCV-2) [1]. *Actinobacillus pleuropneumoniae* is a primary bacterial pathogen and opportunistic bacterial pathogens include *Pasteurella multocida*, *Glaesserella* (*Haemophilus*) *parasuis* and *Streptococcus suis*, among others. The pathogenesis of porcine respiratory disease complex is further complicated by management and environmental factors including overcrowding, ventilation, temperature fluctuations, mixing of animals from different sources, sanitation and high through put [2]. Annual losses (reduced growth rates, lower feed conversion efficiency, death) due to swine respiratory disease in the USA were estimated at USD 664 million in 2011 [3] and divided amongst the breeding herd (45%) and the growing/fattening phase (55%) (prrs.com).

Antimicrobial therapy remains an important pharmaceutical intervention for treatment of infected pigs [4,5]. Pradofloxacin is a third generation dual targeting bactericidal quinolone fluoroquinolone and is the most recently approved for use in companion animals [6] and recently for food animals in April 2024. It has broad spectrum in vitro activity against Gram-positive/negative and “atypical” bacteria (i.e., Chlamydia, Mycoplasma and Mycobacterium species) and some anaerobic organisms [6,7]. Pradofloxacin is a dual targeting drug which simultaneously inhibits DNA gyrase (topoisomerase type II) and topoisomerase IV in susceptible bacteria. This dual targeting characteristic reduces the likelihood for resistance selection from susceptible bacterial populations as an organism would need to simultaneously possess two mutations in the genes encoding for the primary targets thereby conferring resistance. Simultaneous mutations in bacteria from a susceptible bacterial population would be a rare occurrence [8].

New antimicrobial agents are investigated in clinical trials to determine clinical outcomes and by in vitro measurements to determine the spectrum of antimicrobial activity as reported here. These comparisons are usually made with currently approved antimicrobial agents. Susceptibility or resistance determination is by the minimum inhibitory concentration (MIC) where a defined density of bacterial cells (10^5^ cfu/mL) is tested against doubling dilution of the antibiotic, under controlled conditions, and the lowest drug concentration inhibiting growth is the MIC. MIC testing is an internationally accepted measurement in diagnostic laboratories doing such testing [9,10]. A second in vitro measurement called the mutant prevention concentration (MPC) assay determines antimicrobial drug concentrations blocking growth of the least susceptible bacterial cells present in high-density bacterial populations. MPC testing is based on a bacterial density of ≥10^9^ CFUs—a density high enough to account for spontaneous mutant sub-populations that occur over a range of bacterial densities between 10^5^ and 10^9^ CFUs [11,12]. Such high-density bacterial populations are reported to exist in infections of the central nervous system [13], pulmonary [14] and urinary tract [15,16]. Sen et al. reported bacterial populations in skin tissue in excess of 10^5^ CFU/g [17]. As such, high-density bacterial populations exist over a range of infections at different anatomical sites and provide opportunity for resistance selection during antimicrobial therapy. Resistance prevention is a laudable goal of antimicrobial therapy.

From previous in vitro investigations, we reported on the activity of ceftiofur, enrofloxacin, florfenicol, tilmicosin and tulathromycin against swine respiratory bacterial pathogens [18]. In this report, we included the same agents and added pradofloxacin, marbofloxacin and tildipirosin and determined MIC and MPC values for swine isolates of *A. pleuropneumoniae* and *P. multocida* in order to have comparative data for pradofloxacin and the other agents included in this study. Such data have value, as Zhang et al. and Ahmad et al. argued that both MIC and MPC data should be included in pharmacokinetic and pharmacodynamic modelling [19,20]. Pradofloxacin had low MIC values (≤0.016 µg/mL) and correspondingly low MPC values (≤0.016–0.125 µg/mL) with 100% of strains having MPC values at or below the susceptibility breakpoint.

## 2. Results

All bacterial strains were tested to determine the minimum inhibitory concentration (MIC). Strains with susceptibility to all antimicrobial agents—at or below the susceptibility breakpoint—were further investigated to determine the mutant prevention concentration (MPC).

Table 1 is a comparison of the MIC and MPC distribution values for each of the drugs tested against the 30 *A. pleuropneumoniae* strains. The antimicrobial drug concentrations (MIC or MPC) inhibiting 50%, 90% and 100% of the strains tested are shown in the table. Pradofloxacin had the overall lowest MIC values with all strains at ≤0.016 µg/mL followed by marbofloxacin (≤0.03) and ceftiofur (≤0.063). The highest MIC values were seen for tilmicosin and tulathromycin with 28/29 strains having values of ≤8 µg/mL. The modal MIC values with all strains were ≤0.016 µg/mL for ceftiofur and pradofloxacin, 0.031 µg/mL for enrofloxacin and marbofloxacin, 0.25 µg/mL for florfenicol and 4 and 8 µg/mL for tilmicosin and tulathromycin. Pradofloxacin and ceftiofur had the lowest MPC values (≤0.125 µg/mL for all strains) followed by marbofloxacin (≤0.5 µg/mL) and enrofloxacin (≤1 µg/mL). For all strains, florfenicol MPC values were ≤2 µg/mL and ranged from ≤4 to ≥32 µg/mL for tilmicosin and tulathromycin. Modal MPC values were 0.031 µg/mL for pradofloxacin, 0.063 µg/mL for ceftiofur and enrofloxacin, 2 µg/mL for florfenicol and 16 µg/mL for tilmicosin and tulathromycin.

In comparing MIC values, pradofloxacin had lower values than did all the other agents (*p* = 0.008–<0.0001). Ceftiofur had lower MIC values than did enrofloxacin, florfenicol, marbofloxacin, tilmicosin and tulathromycin (*p* < 0.0001 for all comparisons). Enrofloxacin and marbofloxacin had lower MIC values than did florfenicol (*p* < 0.0001 for both comparisons) and enrofloxacin, marbofloxacin and florfenicol had lower MIC values than did tilmicosin and tulathromycin (*p* < 0.0001 for all comparisons). For MPC value comparisons, pradofloxacin had lower MPC values than all other agents (*p* < 0.0001 for all comparisons). Ceftiofur had lower MPC values than all other agents except pradofloxacin (*p* = 0.0036–<0.0001). Enrofloxacin had lower MPC values than did florfenicol, marbofloxacin, tilmicosin and tulathromycin (*p* = 0.008–<0.0001). Marbofloxacin had lower MPC values than did florfenicol, tilmicosin and tulathromycin (*p* < 0.0001 for all comparisons) and tulathromycin had lower MPC values than did tilmicosin (*p* = 0.0129).

Table 2 compares MIC and MPC distribution data for the eight antimicrobial agents tested against the P. multocida isolates. Ceftiofur, enrofloxacin and marbofloxacin had the lowest overall MIC values with all strains inhibited by ≤0.016 µg/mL followed by pradofloxacin at ≤0.031 µg/mL, florfenicol (≤0.5 µg/mL), tulathromycin (≤0.5 µg/mL), tildipirosin (≤2 µg/mL) and finally tilmicosin (≤8 µg/mL). Modal MIC values for ceftiofur, enrofloxacin, marbofloxacin and pradofloxacin were ≤0.016 µg/mL, 0.5 µg/mL for florfenicol and tildipirosin, 2 µg/mL for tilmicosin and 0.25 µg/mL for tulathromycin. Pradofloxacin had the lowest overall MPC values with all strains inhibited by ≤0.063 µg/mL followed by enrofloxacin (≤0.125 µg/mL), marbofloxacin (≤0.25 µg/mL) and ceftiofur (≤0.5 µg/mL). For florfenicol, tildipirosin, tilmicosin and tulathromycin, all strains were inhibited by MPC values ≤ 2 µg/mL, 8 µg/mL, ≥32 µg/mL and 4 µg/mL, respectively. Modal MPC values were ≤0.016 µg/mL for pradofloxacin, 0.125 and 0.5 µg/mL for ceftiofur, 0.063 µg/mL for enrofloxacin and marbofloxacin, 1 µg/mL for florfenicol and tulathromycin, 4 µg/mL for tildipirosin and 8 µg/mL for tilmicosin.

Pradofloxacin, ceftiofur, enrofloxacin and marbofloxacin had lower MIC values than did florfenicol, tildipirosin, tilmicosin and tulathromycin (*p* < 0.0001 for all comparisons). Tulathromycin had lower MIC values than did florfenicol (*p* = 0.0002). Tildipirosin had lower MIC values than tilmicosin (*p* < 0.0001) and tulathromycin had lower MIC values than tildipirosin (*p* < 0.0001). For MPC value comparisons, pradofloxacin had lower MPC values than did all other agents (*p* < 0.0001 for all comparisons). Ceftiofur had lower MPC values than all other agents except pradofloxacin (*p* = 0.0164–<0.0001). Enrofloxacin and marbofloxacin had lower MPC values than florfenicol, tildipirosin, tilmicosin and tulathromycin (*p* < 0.0001 for all comparisons). Tildipirosin had lower MPC values than tilmicosin (*p* < 0.0001) but tulathromycin had lower values than tildipirosin (*p* < 0.0001) and tilmicosin (*p* < 0.0001).

Table 3 summarizes key pharmacokinetic and pharmacodynamic calculations for each of the antimicrobial agents tested. Antimicrobial agents are characterized as time- or concentration-dependent and as such, drug concentration values such as the maximum serum or tissue drug concentration or area under the drug concentration curve are used to determine drug concentration to MIC or MPC ratios for concentration-dependent drugs. Similarly, the time the drug concentration remains above the MIC (and MPC) is important for time-dependent agents. For each calculation, the MIC and MPC values for the strains tested were used along with published values for serum/plasma drug concentrations in swine. As such, values from other publications that differ from the values used/measured in this report will give different calculation results.

## 3. Discussion

Despite variations in studies, methods used, advantages and limitations, Boecters et al. in a systematic review concluded porcine respiratory disease represents a significant economic burden in pig production [35]. Meeuwse and colleagues reported a reduction in mortality in pigs treated with an antimicrobial agent for swine respiratory disease [36]. Strategies for treatment and prevention of *A. pleuropneumoniae* in pigs include antibiotics and vaccines, with current vaccines being minimally protective [37]. For that reason, antimicrobials still play a significant role in the approach to therapy for swine respiratory disease. O’Connor et al. in a systematic review and meta-analysis compared a number of different injectable antibiotic therapies for swine respiratory disease and ranked the various agents based on the likelihood for first therapeutic failure within 5–14 days of initial treatment [38]. From lowest treatment failures to highest the antibiotics were as follows: enrofloxacin, gamithromycin, marbofloxacin, florfenicol, tilmicosin, tulathromycin, amoxicillin, ceftiofur and oxytetracycline. Many of the same antibiotics were included in this in vitro study, and how pradofloxacin would compare to enrofloxacin and marbofloxacin in a similar analysis remains to be determined. The study does show antibiotic efficacy for swine respiratory disease. The authors indicate that while prevention methods are preferred approaches, antibiotic treatment is necessary to ensure the best possible outcome.

In this report, pradofloxacin was compared (in vitro) to several other antimicrobial agents to define MIC and MPC values against a collection of strains that were susceptible to every antimicrobial agent tested. Pradofloxacin had both low MIC (≤0.016–0.031 µg/mL) and MPC values (≤0.016–0.125 µg/mL) against the *A. pleuropneumoniae* and *P. multocida* strains tested. Zhang et al. suggested that potential limitations to MIC testing argues in favour of MPC values being considered in PK/PD modelling [19]. Others have also reported on MPC and PK/PD modelling data [20,39,40,41,42].

As a dual targeting agent, pradofloxacin simultaneously inhibits DNA gyrase and topoisomerase IV—enzymes critical for bacterial DNA replication such that inhibition of these enzymes are lethal to the bacterial cell. Dual targeting is argued to reduce the likelihood for resistance selection from bacterial populations exposed to the drug. Spontaneous mutation occurs over bacterial densities between 10^7^–10^9^ cfu/mL such that a drug targeting a single site would see an elevated MIC as a result of a mutation in the gene encoding the target protein. Bacterial densities in excess of 10^7^ cfu/mL are known to occur in human and animal infections [13,43,44,45,46,47]. Chen and colleagues used a piglet tissue case model to study PK/PD parameters of tilmicosin against *P. multocida* [48]. Only animals with tissue cages with bacterial counts of ≥10^7^ cfu/mL were used for experiments suggesting higher bacterial densities were required for infection. By comparison, for resistance to occur to a dual targeting drug (i.e., pradofloxacin), two simultaneous mutations (one in each gene encoding for the two targets) would need to occur; however, the frequency with which this occurs would be the product of multiplying the mutational frequency (e.g., 1 × 10^−7^–1 × 10^−14^) such that a bacterial population on the magnitude of 10^14^–10^18^ (mutational frequencies of 10^−7^–10^−9^) would be required to have one bacterial cell with two mutations [49]. Such bacterial densities do not occur during animal infections.

Fluoroquinolones are concentration-dependent agents and their antibacterial activity is determined by the maximum serum concentration to MIC (C_max_/MIC) ratio and the area under the curve to MIC (AUC/MIC) ratio. Previously it has been argued that a C_max_/MIC ratio of 8–12 (or higher) and an AUC/MIC > 125 (or higher) are associated with clinical improvement and minimization of resistance selection [50]. The importance of protein binding and the free drug fraction for antimicrobial activity requires consideration [51]. In our study the MIC_90_/MPC_90_ values for pradofloxacin were ≤0.016/0.031 µg/mL for *A. pleuropneumoniae* strains and ≤0.016/0.031 µg/mL for the *P. multocida* strains. Specifically, for pradofloxacin (Table 3) and *A. pleuropneumoniae*, the C_max_/MIC_90_ ratio was 165 and the AUC/MIC_90_ ratio was 1075; for *P. multocida,* 165 and 1075 respectively. Considering MPC values in the calculations, the values for *A. pleuropneumoniae* would be 41.9 and 273, and for *P. multocida* 85.2 and 554.8, respectively. If considering protein binding (33%), the C_max_/MIC_90_ value would be 34.5 and AUC/MIC_90_ values would be 720.3 for both organisms. For C_max_/MPC_90_, the values would be 238.1 and 57.1 for *A. pleuropneumoniae* and *P. multocida,* respectively, and AUC/MPC_90_ would be 182.9 and 371.7, respectively. Pradofloxacin drug concentrations would exceed the mutant selection window for >24 h and as such appears to have a low likelihood for resistance selection. Zhang et al. suggested that limitations in MIC values for PK/PK modelling require consideration of MPC values to potentially impact on antimicrobial resistance [19]. The mutant selection window (MSW) is bordered by the MIC and MPC drug concentrations and Ahmad et al. indicated that dosing to avoid the MSW could slow resistance development [20,52]. For pradofloxacin, the MSW for 90% of strains was between 0.016 and 0.063 µg/mL and below the susceptibility breakpoint and the C_max_ drug concentration was 12× above the MSW, suggesting a low likelihood for resistance selection.

## 4. Materials and Methods

### 4.1. Bacterial Strains

Strains of *A. pleuropneumoniae* (n = 30) and *P. multocida* (n = 41) were generously provided by Purdue University, West Lafayette, IN, USA with organism identification verified by Matrix-assistant laser desorption ionization time of flight (MALDI-TOF) (BioMerieux, St. Laurent, QC, Canada) and by Vitek II. Each strain was stored at −70 °C in skim milk.

### 4.2. Antimicrobial Compounds

The antimicrobial agents used in this study were either purchased commercially (ceftiofur, florfenicol, marbofloxacin, tildipirosin, tilmicosin, tulathromycin) or obtained from the manufacturers (enrofloxacin, pradofloxacin) and prepared/stored in accordance with directions for reconstitution (i.e., sterile water) [53].

### 4.3. MIC Measurements

The recommended Clinical and Laboratory Standards Institute (CLSI) method for MIC susceptibility testing was followed [53]. Bacterial strains were thawed, subcultured twice on blood agar (tryptic soy agar containing 5% sheep red blood cells) (BA) plates (Oxoid, Nepean ON, Canada) for *P. multocida* and chocolate agar plates for *A. pleuropneumoniae* and incubated for 18–24 h at 35–37 °C in room air. Individual bacterial strains were tested by microbroth dilution (×2) containing two-fold drug concentration increments using Mueller–Hinton Broth (MHB) (*P. multocida*) or Mueller–Hinton fastidious broth with yeast extract (MHFBYE) (*A. pleuropneumoniae*) in 96-well microdilution trays. *A. pleuropneumoniae* and *P. multocida* suspension equal to a 0.5 McFarland standard were prepared in sterile saline following which 50 µL was transferred to 5 mL of either MHB (*P. multocida*) or MHFBYE (*A. pleuropneumoniae*) and then 100 µL added to each of the 96 wells to achieve a final inoculum of 5 × 10^5^ cfu/mL. Inoculated trays were incubated for 18–24 h in room air and then examined to determine the MIC—which was recorded as the lowest drug concentration preventing visible growth. Organisms were considered susceptible when the MIC was at or below the susceptibility breakpoint. Five American Type Culture Collection (ATCC) control strains were included for quality control of susceptibility assays: *Staphylococcus aureus* ATCC 29213, *E. coli* ATCC 25222, *Pseudomonas aeruginosa* ATCC 27853, *Enterococcus faecalis* ATCC 29212 and *Histophilus somni* ATCC 700025. MIC values were consistently within acceptable ranges for each control organism/drug.

### 4.4. MPC Measurements

Only strains testing as susceptible (at or below susceptibility breakpoint) by MIC measurements were further tested to determine MPC value (×2). The MPC protocol was adapted from previous methods for *S. pneumoniae*, *A. pleuropneumoniae* and *P. multocida* [18,54]. Starter culture using 5 BA (*P. multocida*) and/or 5 chocolate (*A. pleuropneumoniae)* per isolate were streaked for confluent growth and incubated at 35–37 °C for 18–24 h in room air. Following incubation, the complete contents of all inoculated plates were transferred to 100 mL of MHFBYE (*A. pleuropneumoniae*) or MHB (*P. multocida*), incubated overnight as described and the following day, cultures were estimated to have densities of 3 × 10^8^ cfu/mL by turbidity measurements using a spectrophotomer (Thermo Scientific, Waltham, MA, USA, GENESYSIS IOSVIS). Cultures were subsequently concentrated by centrifugation (5000× *g*) (Beckman Coulter J6−M1 centrifuge) for 30 min at 4 °C and then re-suspended in 3 mL of fresh medium. Drug-containing agar plates had aliquots of 200 µL containing 10^10^ CFU applied to the surface and spread using a sterile L-shaped cell spreader (Fisher Scientific, Ottawa, ON, Canada). Agar dilution plates were prepared by incorporating a drug over a range of doubling dilution concentrations: ceftiofur 0.004–2 µg/mL, enrofloxacin 0.016–2 µg/mL, florfenicol 0.125–32 µg/mL, marbofloxacin 0.008–2 µg/mL, tildipirosin 1–128 µg/mL, tilmicosin 0.5–64 µg/mL and tulathromycin 0.25–16 µg/mL. Following incubation in room air, plates were examined for growth at 24 h then re-incubated for an additional 24 h and re-examined. The lowest drug concentration preventing any visible growth after 48 h incubation was recorded as the MPC.

### 4.5. Statistical Analysis

MIC and PC values for each drug were compared between drugs using the Exact Wilcoxon test. For the purpose of statistical analysis, values of ≤0.016 or ≥32 were changed to 0.016 and 32. A *p* value ≤ 0.05 was considered statistically significant.

## 5. Conclusions

Pradofloxacin is the newest veterinary fluoroquinolone and the newest antimicrobial agent to be approved for use in food animals and specifically for swine with respiratory disease. Pradofloxacin had low MIC and MPC values and favorable PK/PD ratios (even when considering protein binding) considering C_max_/MIC_90_, C_max_/MPC_90_, AUC/MIC_90_ and AUC/MPC_90_—values associated with improved clinical outcome and minimization of resistance selection. Having such data as reported here will help with PK/PD modelling. To our knowledge, this represents the most comprehensive comparison of these agents against swine respiratory pathogens and the first report of such for pradofloxacin and tildipirosin.

As a dual targeting fluoroquinolone, pradofloxacin has a low likelihood for resistance selection. Additionally, data on the frequency of plasmid-mediated resistance to fluoroquinolones amongst *A. pleuropneumoniae* and *P. multocida* strains from swine are lacking, however, Ma et al. indicated that target site mutations are the major mechanism of quinolone resistance. As such, pradofloxacin should be a welcomed addition to antimicrobials useful for treating swine respiratory disease.

## Figures and Tables

**Table 1:** MIC and MPC distribution for 29 *Actinobacillus pleuropneumoniae* strains tested against 7 antimicrobial agents.

Drug	Bacteriostatic(S)/ Bactericidal(C)	MIC/MPC Distribution Values (µg/mL)													
		<0.016	0.031	0.063	0.125	0.25	0.5	1	2	4	8	16	≥32		
		**MIC**												**MIC** **Breakpoint**	**MIC_50/90/100_**
Ceftiofur	C	19	9	1	_	_	_	_	_	_	_	_	_	≤2	≤0.016/0.031/0.063
Enrofloxacin	C	2	23	3	_	1		_	_	_	_	_	_	≤0.25	0.031/0.031/0.25
Florfenicol	S	_	_	_	1	26	2	_	_	_	_	_	_	≤2	0.25/0.25/0.5
Marbofloxacin	C	3	25	_	1	_	_	_	_	_	_	_	_	N/A	0.031/0.031/0.125
Pradofloxacin	C	29	_	_	_	_	_	_	_	_	_	_	_	≤0.125 ***	≤0.016/≤0.016/≤0.016
Tilmicosin	S	_	_	_	_	_	_	_	1	16	11	1		≤16	4/8/≥32
Tulathromycin	S	_	_	_	_	_	_	_	1	12	15	1 *	_	≤64	8/8/≥16
		**MPC**													**MPC_50/90/100_**
Ceftiofur		2	7	19	1	_	_	_	_	_	_	_	_	_	0.063/0.063/0.125
Enrofloxacin		_	_	10	3	6	7	3	_	_	_	_	_	_	0.25/0.5/1
Florfenicol		_	_	_	_	_	_	1	28	_	_	_	_	_	2/2/2
Marbofloxacin		_	3	17	5	2	2	_	_	_	_	_	_	_	0.063/0.25/1
Pradofloxacin		12	13	1	3	_	_	_	_	_	_	_	_	_	0.031/0.063/0.125
Tilmicosin		_	_	_	_	_	_	_	_	3 **	1	19	6	_	16/≥32/≥32
Tulathromycin		_	_	_	_	_	_	_	_	7	5	9	8	_	16/≥32/≥32

MIC = minimum inhibitory concentration; MPC = mutant prevention concentration. * ≥16 µg/mL; ** ≤ 4 µg/mL; *** based on breakpoint for *P. multocida*. MIC_50/90/100_—drug concentration inhibiting 50%, 90% and 100% of strains tested. MIC Breakpoint—drug concentration for which an MIC at or below this value is considered susceptible.

**Table 2:** MIC and MPC distribution values for 41 *P. multocida* strains tested against 8 antimicrobial agents.

Drug	Bacteriostatic(S)/ Bactericidal(C)	MIC/MPC Distribution Values (µg/mL)													
		<0.016	0.031	0.063	0.125	0.25	0.5	1	2	4	8	16	≥32		
		**MIC**												**MIC** **Breakpoint**	**MIC_50/90/100_**
Ceftiofur	C	41												≤2	≤0.016/≤0.016/≤0.016
Enrofloxacin	C	41												≤0.25	≤0.016/≤0.016/≤0.016
Florfenicol	S					18	23							≤2	0.5/0.5/0.5
Marbofloxacin	C	41												NA	≤0.016/≤0.016/≤0.016
Pradofloxacin	C	38	3											≤0.125	≤0.016/≤0.016/0.031
Tildipirosin	S			1	1	5	14	10	10					≤4	0.5/2/2
Tilmicosin	S						2	2	20	15	2			≤16	2/4/8
Tulathromycin	S				8	24	9							≤16	0.25/0.5/0.5
		**MPC**													**MPC_50/90/100_**
Ceftiofur		3	8	5	12	12	1								0.125/0.25/0.5
Enrofloxacin		4	11	15	11										0.063/0.125/0.125
Florfenicol							3	37	1						1/1/2
Marbofloxacin		4	10	13	12	2									0.063/0.125/0.25
Pradofloxacin		22	18	1											≤0.016/0.031/0.063
Tildipirosin								2	8	28	3				4/4/8
Tilmicosin									2	8	20	7	4		8/16/≥32
Tulathromycin							10	27	2	2					1/1/4

MIC = minimum inhibitory concentration; MPC = mutant prevention concentration. MIC_50/90/100_—drug concentration inhibiting 50%, 90% and 100% of strains tested. MIC Breakpoint—drug concentration for which an MIC at or below this value is considered susceptible.

**Table 3:** Pharmacokinetic and pharmacodynamic values for 8 antimicrobial agents tested * [21,22,23,24,25,26,27,28,29,30,31,32,33,34].

Compound	C_max_	T_issuemax_	AUC_24_	C_max_/MIC_90_	C_max_/MPC_90_	AUC_24_/MIC_90_	AUC_24_/MPC_90_	T > MIC_90_	T > MPC_90_	% Protein Binding * (µg/mL)	Concentration (C) or Time (T) Dependent
***A. pleuropneumoniae***											
Ceftiofur	23.3	5.8	358	751.6	369.8	11,548.4	5682.5	>24 h	>24 h	>90	T > MIC
Enrofloxacin	1.1	4.6	47.86	35.48	2.2	1543.9	95.7	>24 h	>24 h	31	AUC/MIC, C_max_/MIC
Florfenicol	4.5	2.94	64.9	18	2.1	259.6	32.5	>24 h	~10 h	~15	T > MIC
Marbofloxacin	1.6	NA	31.17	48.4	6.4	1005.5	124.7	>24 h	>24 h	<10	AUC/MIC, C_max_/MIC
Pradofloxacin	2.64	0.81	17.2	165	41.9	1075	273	>24 h	>24 h	33	AUC/MIC, C_max_/MIC
Tildipirosin	0.767	14.77	21 ^a^							30	AUC/MIC
Tilmicosin	1.67	NA	34.86	0.03	0.008	4.4	2.6	0	0	~15	T > MIC
Tulathromycin	0.6	3.2	122	0.07	0.019	15.3	3.8	0	0	~40	T > MIC
***P. multocida***											
Ceftiofur	23.3	5.8	358	1456.3	93.2	22375	1432	>24 h	>24 h		
Enrofloxacin	1.1	4.6	47.86	68.75	8.8	2991.3	382.9	>24 h	>24 h		
Florfenicol	4.5	2.94	64.9	9	4.5	129.8	64.9	>24 h	~20 h		
Marbofloxacin	1.6	NA	31.17	100	12.8	1948.1	249.4	>24 h	>24 h		
Pradofloxacin	2.64	0.81	17.2	165	85.2	1075	554.8	>24 h	>24 h		
Tildipirosin	0.767	14.77	21	0.38	0.19	10.5	5.25	0	0		
Tilmicosin	1.67	NA	34.86	0.21	0.1	8.7	2.4	0	0		
Tulathromycin	0.6	3.2	122	1.2	0.6	244	122	~9 h	0		

* Protein binding is not considered in the calculations. ^a^ AUC based on cattle (similar value for goats). MIC = minimum inhibitory concentration; MPC = mutant prevention concentration; C_max_ = maximum serum concentration; T_issuemax_ = maximum tissue concentration; AUC = area under the curve; N/A = not available.

## Data Availability

The data that support the findings of this study are available from the corresponding author upon reasonable request.
